# Repetitive Architecture of the *Haemophilus influenzae* Hia Trimeric Autotransporter

**DOI:** 10.1016/j.jmb.2008.09.085

**Published:** 2008-12-26

**Authors:** Guoyu Meng, Joseph W. St. Geme, Gabriel Waksman

**Affiliations:** 1 Institute of Structural and Molecular Biology at UCL/Birkbeck, Malet Street, London WE1C 7HX, UK; 2 Departments of Pediatrics and Molecular Genetics and Microbiology, Duke University Medical Center, Durham, NC 27710, USA

**Keywords:** MAD, multiwavelength anomalous dispersion, PEG, polyethylene glycol, ESRF, European Synchrotron Radiation Facility, Hia, autotransporter, adhesin, structural biology, *Haemophilus influenzae*

## Abstract

The Hia autotransporter of *Haemophilus influenzae* belongs to the trimeric autotransporter subfamily and mediates bacterial adherence to the respiratory epithelium. In this report, we show that the structure of Hia is characterized by a modular architecture containing repeats of structurally distinct domains. Comparison of the structures of HiaBD1 and HiaBD2 adhesive repeats and a nonadhesive repeat (a novel fold) shed light on the structural determinants of Hia adhesive function. Examination of the structure of an extended version of the Hia translocator domain revealed the structural transition between the C-terminal translocator domain and the N-terminal passenger domain, highlighting a highly intertwined domain that is ubiquitous among trimeric autotransporters. Overall, this study provides important insights into the mechanism of Hia adhesive activity and the overall structure of trimeric autotransporters.

## Introduction

*Haemophilus influenzae* is a Gram-negative bacterium that causes disease exclusively in humans. Nonencapsulated (nontypeable) strains are responsible for most *H. influenzae* localized respiratory tract diseases such as otitis media, sinusitis, and pneumonia, and encapsulated strains account for most *H. influenzae* invasive diseases, including sepsis and meningitis.^1^

The pathogenesis of *H. influenzae* disease begins with colonization of the upper respiratory tract,^2,3^ followed by either contiguous spread within the respiratory tract or invasion of the bloodstream. The process of colonization requires bacterial adherence to the respiratory epithelium and is facilitated by both pilus and nonpilus adhesive factors.^4^ In a subset of nontypeable strains, the major nonpilus adhesin is a protein called Hia.^5^ Among encapsulated strains, the primary nonpilus adhesin is a homolog of Hia called Hsf.^6^

Both Hia and Hsf belong to the expanding autotransporter family of proteins and are examples of so-called trimeric autotransporters, which are characterized by a trimeric architecture with three identical subunits.^7–10^ Autotransporters are synthesized as precursor proteins with three functional domains, namely, an N-terminal signal peptide, an internal passenger domain, and a C-terminal pore-forming translocator domain.^11,12^ The C-terminal translocator domain is embedded in the outer membrane and facilitates delivery of the internal passenger domain to the bacterial surface. In conventional autotransporters, the C-terminal translocator domain contains approximately 300 amino acids and is monomeric.^9,13^ In contrast, in trimeric autotransporters, the translocator domain contains ∼ 60–70 amino acids and forms trimers in the outer membrane.^7,14^ In a recent work, Oomen *et al.* solved the crystal structure of the translocator domain of the *Neisseria meningitidis* NalP conventional autotransporter and observed a monomeric β-barrel pore containing 12 transmembrane β-strands and traversed by an N-terminal α-helix.^15^ More recently, we solved the structure of the Hia translocator domain and found that it forms a β-barrel with 12 transmembrane β-strands as well, in this case with each subunit in the trimer contributing four strands. The Hia β-barrel has a 1.8-nm-diameter central channel that is traversed by three N-terminal α-helices, one from each subunit.^16^

A number of models have been proposed for the translocation of autotransporter passenger domains across the outer membrane.^13,15^ However, only two of these models are consistent with the structures of the translocator domains of NalP and Hia, namely, the “hairpin” model and the “Omp85” model.^15,16^ According to the hairpin model, translocation of the passenger domain is initiated with the C-terminal end of the passenger domain forming a hairpin structure in the β-barrel and is completed as the rest of the passenger domain sequence slides through the pore. According to the Omp85 model, the pore-forming Omp85 outer-membrane protein facilitates insertion of the autotransporter translocator domain into the outer membrane and then translocates the autotransporter passenger domain through the Omp85 pore.^15^

Hia contains two homologous binding domains, called HiaBD1 and HiaBD2, that interact with the same host cell receptor. The nature and identity of the receptor(s) are still unknown. In the prototype Hia protein in *H. influenzae* strain 11, HiaBD1 is defined by amino acids 585–705 and binds to cultured epithelial cells with a *K*_d_ of 0.05–0.1 nM, whereas HiaBD2 is defined by amino acids 51–166 and binds to cultured epithelial cells with a *K*_d_ of 1–2 nM.^17^ In a previous work, we determined the crystal structure of HiaBD1 and established that perturbation of subunit–subunit interactions results in disruption of the trimer and loss of adhesive activity.^18^ The receptor-binding pocket is formed by an acidic patch that is present on all three faces of the trimeric domain, providing potential for a multivalent interaction with the host cell surface.^18^ Although the structure of HiaBD2 is not known, a structure-based sequence alignment identified differences in residue compositions in the acidic patch that are likely to be responsible for the receptor-binding affinity lower than that for HiaBD1.^18^ Work on the Hsf protein has identified two binding domains homologous to HiaBD1 and HiaBD2, called HsfBD1 and HsfBD2, respectively.^8^ Based on site-directed mutagenesis, the receptor-binding pockets in HsfBD1 and HsfBD2 are formed by acidic patches that parallel the acidic patches in HiaBD1 and HiaBD2.^8^

In the current study, we show that the architecture of Hia and Hsf is characterized by passenger domains with multiple repeats of primarily three distinct structural domains. In addition, we compare the structures of two structural repeats with adhesive activity and one structural repeat with nonadhesive activity, shedding light on the structural determinants of Hia and Hsf adherence. We also report the structure of an extended version of the Hia translocator domain, revealing the structural transition between the translocator domain and the N-terminal passenger domain. This transition is mediated by a highly intertwined domain that is ubiquitous among trimeric autotransporters. Thus, the structures of Hia fragments presented in this report reveal a remarkable repetitive architecture in trimeric autotransporters.

## Results

### Domain arrangement in Hia-like adhesins

**Fig. 1:**
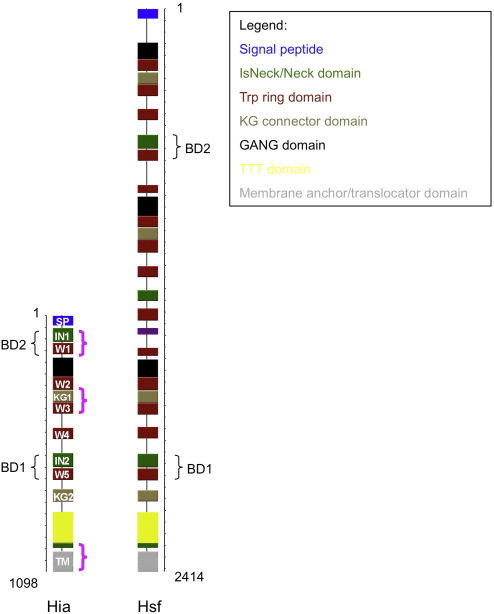
The repetitive domain arrangement of nonencapsulated *H. influenzae* Hia and encapsulated *H. influenzae* Hsf. The sequences in Hia that are determined crystallographically in this report are highlighted with purple brackets.

To gain insight into the structure of the full-length Hia protein, the Hia amino acid sequence was submitted to the daTAA server,† a server designed to analyze sequences of trimeric autotransporters.^19^ As shown in Fig. 1, the sequence analysis predicted an impressive multirepetitive domain arrangement (Fig. 1) consisting of five domain types, with some repeated several times along the sequence. The Hia sequence contains five so-called “Trp-ring” domains (residues 117–166, 263–319, 372–421, 481–530, and 654–705, designated W1, W2, W3, W4, and W5, respectively, in Fig. 1) and three so-called “Neck” domains, including two domains with functional insertions (referred to as “IsNeck” domains; residues 53–113, 589–650, and 975–996, designated IN1, IN2, and Neck, respectively, in Fig. 1). In addition, there are two so-called “KG” domains (residues 320–372 and 743–798, designated KG1 and KG2, respectively, in Fig. 1), one so-called “GANG” domain (residues 180–263), one so-called “TTT” domain (residues 842–974), one signal peptide (residues 1–49, designated SP in Fig. 1), and one membrane anchor/translocator domain (residues 1022–1098, designated “TM” in Fig. 1). The distinctive features of the Trp-ring, Neck, IsNeck, KG, GANG, and TTT domains are well described‡.^19^

A similar analysis of the *H. influenzae* Hsf adhesin predicted a repetitive domain arrangement analogous to the arrangement in Hia, including 14 Trp-ring repeats, 4 Neck domains (with 3 IsNeck domains), 4 KG domains, 3 GANG domains, 1 TTT domain, a signal peptide, and a membrane anchor/translocator domain (Fig. 1).

The adhesive activities in both Hia and Hsf locate to regions containing one IsNeck domain, followed by one Trp-ring domain (Fig. 1). Interestingly, not all IsNeck and Trp-ring domains are adhesive. Instead, there appears to be a requirement for immediately contiguous IsNeck and Trp-ring domains—an arrangement that is achieved in only two regions in Hia (HiaBD1, formed by IN2 and W5, and HiaBD2, formed by IN1 and W1) and two regions in Hsf (HsfBD1, formed by IN3 and W14, and HsfBD2, formed by IN1 and W4) (Fig. 1).

### Structures of Hia_51–166_ and Hia_307–422_

Trp-ring domains appear to be crucial repeated modular units in Hia, both in the general architecture of the passenger domain and in the structure of the binding domains. However, the sequences of these domains are very poorly conserved, and it is not clear whether they share the same structural fold. To investigate this issue and to clarify the structural determinants of Hia-mediated binding, we determined the structures of two Hia passenger domain fragments: one with adhesive activity (Hia_51–166_, corresponding to HiaBD2, which shares a 45% sequence identity with HiaBD1), and the other with no adhesive function (Hia_307–422_, which shares an 18% sequence identity with HiaBD1).

**Fig. 2:**
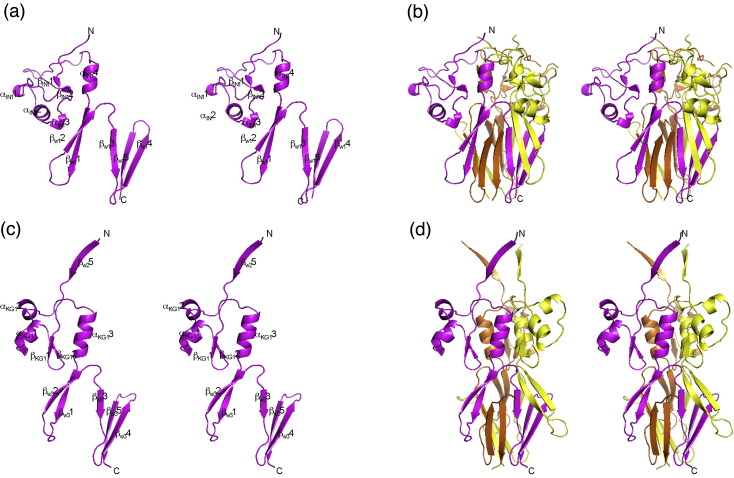
Structures of the Hia_51–166_ and Hia_307–422_ monomers and trimers. (a) Stereo ribbon diagram of the Hia_51–166_monomer. Secondary structures, including helices and strands, are labeled. (b) Stereo ribbon diagram of the Hia_51–166_ trimer. The three subunits are shown in magenta, yellow, and orange. (c) Stereo ribbon diagram of the Hia_307–422_ monomer. Secondary structures, including helices and strands, are labeled. (d) Stereo ribbon diagram of the Hia_307–422_ trimer. The three subunits are shown in the same color scheme as mentioned above.

**Fig. 3:**
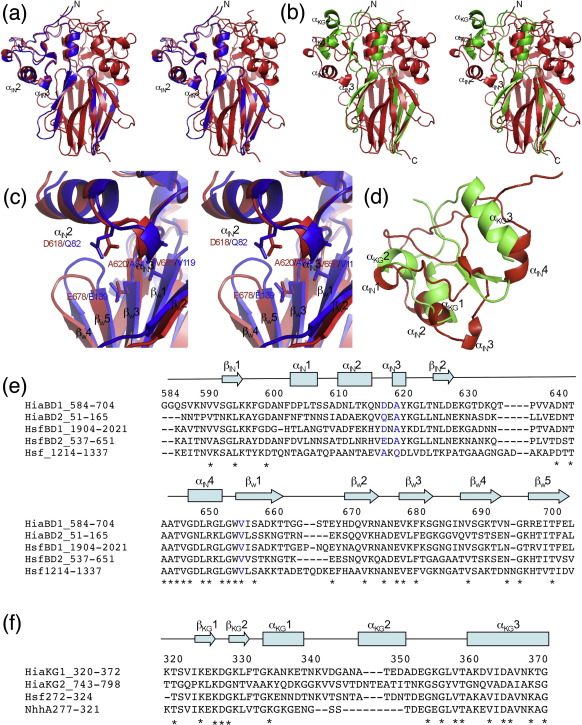
Structure comparison between adhesive domains HiaBD1 and HiaBD2 and the nonadhesive domain Hia_307–422_. (a) Stereo ribbon diagram of the superimposed HiaBD1 (red) and HiaBD2 (blue) domains. (b) Stereo ribbon diagram of the superimposed HiaBD1 (red) and Hia_307–422_ (green) domains. (c) Detailed molecular feature of the groove formed by the base of the IN domain (α_IN_2 and α_IN_3) and the top of the adjacent W domain (β_W_1 and β_W_3). Mutations of residues D618 (in the loop between α_IN_2 and α_IN_3), A620 (in α_IN_3), and V656 (in βW1) all abolished the adhesion activities of HiaBD1 *in vivo* and *in vitro*. (d) Ribbon diagram of the superimposed IN2 (red) and KG1 (green) domains. The helices, including α_KG_1, α_KG_2, and α_KG_3 in KG domain and α_IN_2, α_IN_1, and α_IN_4 in IN domain, are labeled respectively. (e) Sequence alignment of the IN and W adhesive-like domains among Hia/Hsf adhesins. (⁎) Invariant residues. The three functional important residues are highlighted in blue. (f) Sequence alignment of the KG domains between Hia/Hsf/NhhA.

The structure of HiaBD2 was determined to a resolution of 2.0 Å, using molecular replacement and HiaBD1 as search model (Tables 1 and 2; Supplementary Fig. 1). HiaBD2 contains two well-defined structural domains, namely, IN1 and W1. IN1 is a globular knob-like domain with a simple α/β sandwich fold. Two short β-strands (β_IN1_1 and β_IN1_2) form a short β-sheet, flanked by four helices: α_IN1_1–α_IN1_3 on one side and α_IN1_4 on the other side (Fig. 2a). In the trimer, α_IN1_4 from each HiaBD2 subunit forms a three-helix bundle parallel with the axis of the trimer from which the three knob-like shapes protrude laterally (Fig. 2b). The W1 domain of HiaBD2 is an all-β domain consisting of five long β-strands (β_W1_1–β_W1_5; Fig. 2a). In the monomer, these strands—including two β-hairpins (β_W1_1–β_W1_2 and β_W1_4–β_W1_5) and a connector strand (β_W1_3)—are remarkably segregated and twisted into an N-shape (Fig. 2a). In the trimer, these strands are highly intertwined, forming a five-stranded β-sheet on each face of the trimer and serving as a surface against which the α_IN1_2 and α_IN1_3 helices of IN1 rest (Fig. 2b). The structures of HiaBD2 and HiaBD1 are very similar, aligning with a root mean square deviation (RMSD) in C^α^ positions of 1.1 Å between IN1 of HiaBD2 and IN2 of HiaBD1, and 1.5 Å between W1 of HiaBD2 and W5 of HiaBD1 (see superposition of the two structures in Fig. 3a).

For Hia_307–422_, the initial phasing efforts using either HiaBD1 or HiaBD2 as search model for molecular replacement were unsuccessful. Thus, the Ile residue at position 324 in the nonconserved region of KG1 was mutated to Met, allowing production of selenomethionine-derivatized crystals usable for phasing by the multiwavelength anomalous dispersion (MAD) method.^18^ The crystal structure of Hia_307–422_ was determined to a resolution of 1.8 Å.

The nonadhesive Hia_307–422_ fragment contains three distinct domains, including the last strand of the preceding W2 domain (β_W2_5), a novel “KG1” domain, and the W3 domain (Fig. 2c). β_W2_5 lies in the crystal packing interface between two Hia_307–422_ trimers and forms an anti-parallel β-sheet with the adjacent β_W2_5 strand of a separate trimer. Although the conformation of β_W2_5 observed in Hia_307–422_ might not accurately reflect the conformation it adopts in the W2 domain, it may be used to envisage the structural transition between Trp-ring domains via a KG connector domain.

The structure of the KG1 domain appears to be related to the structure of the IsNeck domain, with the secondary structures positioned similarly relative to each other. Indeed, superposition of IN2 of HiaBD1 onto KG1 of Hia_307–422_, obtained by aligning the two-stranded β-sheets of both domains (Fig. 3d), shows that the α_KG1_1, α_KG1_2, and α_KG1_3 helices are equivalent to the α_IN2_2, α_IN2_1, and α_IN2_4 helices, respectively. In both IN2 and KG1, the C-terminal helix forms an important element of the trimer interface (Fig. 2d). This structural similarity was not anticipated from sequence alignment. However, it must be noted that the topologies of the IN and KG domains(i.e., the way the secondary structures are connected) are very different (Figs. 2a and c and 3e and f).

The W3 domain in Hia_307–422_ is similar to the W1 domain in HiaBD2 and to the W5 domains in HiaBD1, with the same number of strands and the same angle of the strands relative to the axis of the trimer (Fig. 3a). All of these strands twist and intertwine into a functional trimer, with a total buried area between the subunits well over 8000 Å^2^ (Fig. 2b and d).

### Structure comparison between Trp-ring domains

**Fig. 4:**
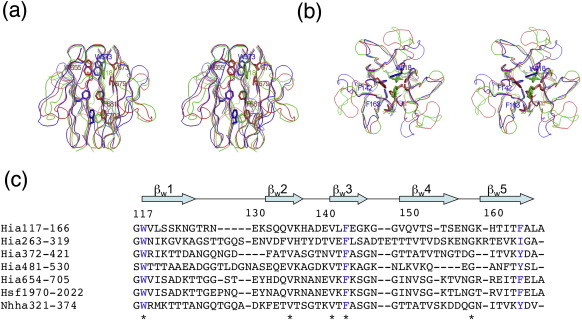
Structure comparison between Trp-ring domains. (a) Stereo ribbon diagram of the superimposed W1 (blue), W3 (green), and W5 (red) domains viewed from the side. (b) Stereo ribbon diagram of the superimposed Trp-ring domains viewed from the top. (c) Sequence alignment of the Trp-ring domains between Hia/Hsf/NhhA. In (a) and (b), the conserved residues at the trimeric interface are shown in stick representation. In (c), these residues are shown in blue.

Comparison of all of the available Trp-ring domain structures of Hia, namely, W1 (residues 117–166), W3 (residues 372–421), and W5 (residues 654–705), reveals only minor differences (Fig. 4). The RMSDs of the main-chain atoms of these Trp-ring domains are 1.6 Å between W1 and W3, 1.7 Å between W3 and W5, and 1.5 Å between W1 and W5. The major variations among these structures lie in the β_W_1–β_W_2, β_W_3–β_W_4, and β_W_4–β_W_5 loops away from the axial hydrophobic cavity of the trimer (Fig. 4). Overall, the most highly conserved residues map at the trimeric interface (Fig. 4b and c). Two invariant aromatic residues in the β_W_1 and β_W_3 strands of the W1, W3, and W5 Trp-ring domains (W118/W373/W655 and F142/F398/F681, respectively) are found in immediate trimeric contact in the upper part of the Trp-ring domain. Another two invariant Val residues in the β_W_2 and β_W_3 strands (V134/V390/V673 and V140/V396/V679) also map to this region and could play a supportive role underneath the invariant Trp residues (Fig. 4c). Finally, a third less conserved aromatic patch in the β_W_5 strand is clearly visible in the lower part of the W1, W3, and W5 domains (F163/Y419/F702) (Fig. 4). The only W domain in which this aromatic ring is not seen is W2, where the trimeric interface is mediated by Ile residues (Fig. 4c).

### Structure comparison between HiaBD1 and HiaBD2 adhesive domains and the Hia_307–422_ nonadhesive domain

In our previous report of the HiaBD1 structure, we identified the surfaces responsible for binding activity (Fig. 3c). This region is located in a groove formed by the base of the IN domain (α_IN_2 and α_IN_3) and the top of the W domain (β_W_1 and β_W_3). Notably, individual mutations of residues D618 (in the loop between α_IN_2 and α_IN_3), A620 (in α_IN_3), and V656 (in β_W_1) abolished the adhesive activity of HiaBD1 (Fig. 3c and e).^18^

While HiaBD1 has structural similarities with Hia_307–422_, α_IN_3 in HiaBD1 does not have a corresponding region in KG1 in Hia_307–422_, and α_IN_2 in HiaBD1 is oriented differently from α_KG_1 in Hia_307–422_ (Fig. 3b and d). Thus, two important structural determinants of binding in the IN domain are either absent or misoriented in the KG domain, explaining why Hia_307–422_ is nonadhesive.

In an earlier work, we found that HiaBD1 and HiaBD2 recognize the same host cell receptor but with different binding affinities.^17^ However, due to the lack of structural information on HiaBD2, it was not clear which residues account for this difference in adhesive activity. The examination of the binding groove described above (Fig. 3c and e) reveals only one notable difference among functionally important residues between HiaBD1 and HiaBD2 in this region, namely, D618 in HiaBD1 corresponds to a glutamine residue in HiaBD2, resulting in a change in charge and a longer side chain. The corresponding residues in HsfBD1 and HsfBD2 are aspartic acid and glutamic acid, respectively. HsfBD1 and HsfBD2 interact with the same host cell receptor recognized by HiaBD1 and HiaBD2, with HsfBD1 exhibiting a binding affinity higher than that of HsfBD2,^8^ suggesting that the extended side chain of glutamic acid may result in steric hindrance (Fig. 3e). Interestingly, Hsf_1214–1337_ is a region that contains an IsNeck domain and a Trp-ring domain—an arrangement that appears to determine adhesive function. However, in Hsf_1214–1337_, the residues corresponding to D618 and A620 in HiaBD1 are Ala and Gln (Fig. 3e). Consistent with the information on the binding pockets in HiaBD1, HiaBD2, HsfBD1, and HsfBD2, Hsf_1214–1337_ lacks adhesive activity.^8^

### Transmembrane structure of Hia_973–1098_

Although the coding sequence used for the expression and purification of the Hia translocator domain contained residues 937–1098, only the last 126 residues were visible in the electron density map (Hia_973–1098_). The structure was determined to a resolution of 3.0 Å.

**Fig. 5:**
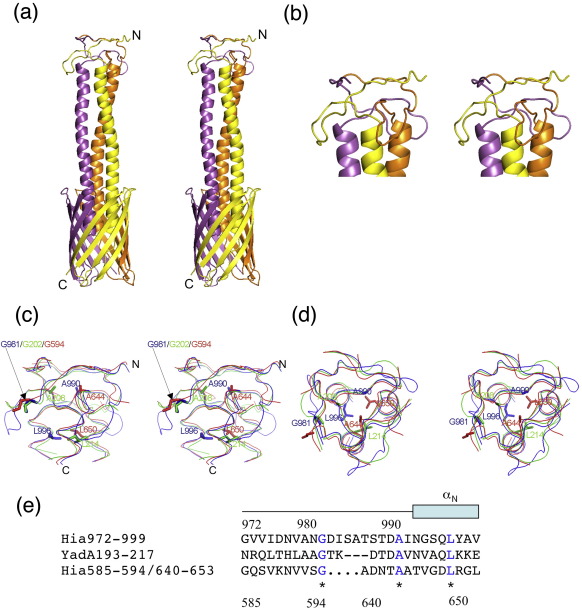
Transmembrane structure of Hia_973–1098_. (a) Stereo ribbon diagram of the Hia_973–1098_ trimer. The three subunits are shown in magenta, yellow, and orange. (b) Stereo ribbon diagram of the Hia_Neck_ domain. (c) Stereo ribbon diagram of the superimposed domains Hia_Neck_ (blue), Neck in IN2 (red), and YadA_Neck_ (green) viewed from the side. (d) Stereo ribbon diagram of the same superimposed Neck domains viewed from the top. (e) Sequence alignment of the Neck domains between Hia_Neck_, Neck in IN2, and YadA_Neck_.

Compared to the previously published Hia_998–1098_ structure, the structure of Hia_973–1098_ reveals the full extent of the helices passing through the β-barrel and an extra highly intertwined Neck domain (Hia_Neck_) sitting on top (Fig. 5a and b). Neck domains are short connector domains (∼ 20–30 residues; Fig. 5e) between α-helical coiled coils and β-regions^18,20^ that are often interrupted by the insertion of sequences to form IsNeck domains.^19,21^

The Hia_973–1098_ trimer is formed through juxtaposition of the transmembrane β-sheets (residues 1043–1098), stacking of the long α-helices (residues 992 and 1036), and interactions between the long loops of the Neck domain along a 3-fold axis running through the center of the trimeric structure (residues 972 and 991) (Fig. 5a). The transmembrane parts of Hia_973–1098_ are virtually the same as those in Hia_998–1098_. The three α-helices protrude from the 12-stranded β-barrel and form a three-helix coiled coil. The last residues in all three helices introduce a 90° kink, followed by an extended hairpin loop. There are only a few intramolecular contacts within Hia_Neck_; thus, the Neck domain appears to hold together via its neighboring subunits through side-chain, van der Waals, and main-chain hydrogen-bonding interactions (Fig. 5c and d). Comparison of Hia_Neck_ with the necks of Hia_IN2_ and YadA (YadA_Neck_) shows that all three are very similar. The RMSDs between the main-chain atoms of these Neck-like domains are 1.5 Å between Hia_Neck_ and Hia_IN2_, 2.5 Å between Hia_IN2_ and YadA_Neck_, and 1.0 Å between Hia_Neck_ and YadA_Neck_. Structure-based sequence alignment reveals three invariant residues in Hia_Neck_, YadA_Neck_, and Hia_IN2_, including G981/G202/G594, A990/A208/A644, and L996/L214/L650 (Fig. 5c–e). G981/G202/G594 lies at the turn of the hairpin-like loops away from the axis of the overall trimeric structure and does not contribute any van der Waals or hydrogen-bonding interactions. Interestingly, these glycines are also the sites of major sequence insertions in the IsNeck domains. A990/A208/A644 and L996/L214/L650 always map axially in the trimeric packing interface, playing important roles in holding the trimeric Neck domain together.

## Discussion

Following determination of the structures of HiaBD1^18^ and the Hia translocator domain,^16^ it became clear that trimeric autotransporters share specific structural features and also have distinct structural differences compared with conventional autotransporters. Among the similarities is the translocator domain, which consists of a conserved 12-stranded β barrel with a central channel traversed by one or three α helices. Among the distinguishing features is the highly singular fold of the adhesive domains of trimeric autotransporters, where the fold of each of the subunits can be understood only in the context of the trimer due to the highly intertwined nature of the trimeric interface.

**Fig. 6:**
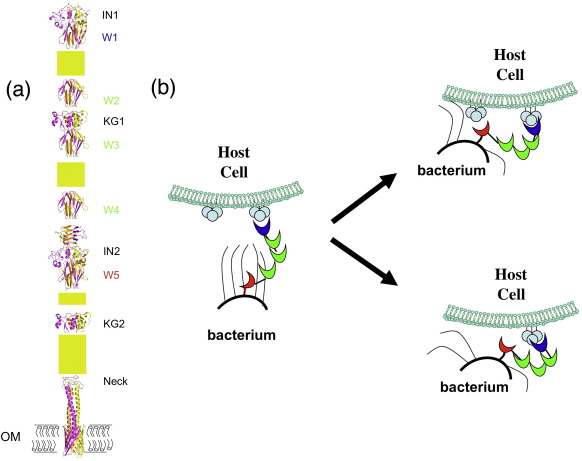
Modular architecture of Hia and putative two-step adhesive mechanism. (a) Modular architecture of Hia adhesin. While the structures of IN1, W1, KG1, W3, IN2, W5, Neck, and transmembrane anchor/translocator domains are determined crystallographically, W2/W4 and KG2 are modeled based on W1/W3/W5 and KG1, respectively. Yellow rectangles represent the Hia sequences that are yet to be structurally characterized, including the predicted N-terminal GANG and C-terminal TTT domains. (b) A putative two-step adhesive mechanism utilized by Hia/Hsf-like adhesin to form an intimate association between the bacterium and the host cell. The adhesive Trp-ring domains with different binding capacities are shown in blue and red. The nonadhesive Trp-ring domains are shown in green.

In this study, we identified novel distinguishing features of Hia. In particular, the Hia passenger domain consists of repeats of structurally similar domains, including five structurally identical Trp-ring domains strung together by two IsNeck domains and two KG domains (Fig. 6). Furthermore, we found that the IsNeck domains and the KG domains share a similar three-dimensional organization, although they differ topologically. At the C-terminus, the multidomain structure of the passenger domain is connected to the Hia membrane anchor/translocator domain via an α-helical coiled-coil-like structure preceded by a Neck connector domain.

While the Neck domains appear to be highly conserved in trimeric autotransporters (particularly at the junction of the passenger and translocator domains), Trp-ring and IsNeck domains appear to be present in only a few of these proteins, and KG domains are even less common (Supplementary Fig. 2). Interestingly, Trp-ring and IsNeck domains appear to be linked evolutionarily, as there are no known trimeric autotransporters exhibiting one without the other. This feature may be related to the observation that both domains must be juxtaposed to display adhesive activity (although this arrangement is not sufficient for adhesive activity, as highlighted by Hsf_1214–1337_).

The implications of such a modular architecture are twofold. First, while conventional autotransporters adopt a relatively simple β-helical arrangement, the folding of trimeric autotransporters is much more complex, not only requiring simultaneous processing of all three subunits but also following a complex folding pathway involving multiple structural domains of different types. Second, the modular architecture of trimeric autotransporters may provide a versatile structural template that can be utilized to create new isoforms by gene shuffling, duplication, or recombination. Hsf may be a good illustration of this concept. The Hsf adhesin includes 14 Trp-ring repeats chained together by three IsNeck domains, four KG domains, and three GANG domains. However, the domain organization at the C-terminus of the Hsf passenger domain is remarkably similar to the organization of Hia, while the N-terminus of Hsf resembles NhhA, an *N. meningitidis* trimeric autotransporter. Thus, Hsf could have arisen from duplication of the Hia gene and capture of an NhhA-like gene (Supplementary Fig. 2). A similar chimeric architecture has been proposed for the head domain of the trimeric autotransporter BadA from *Bartonella henselae*.^22^

Both Hia and Hsf contain two binding domains (HiaBD1/HsfBD1 and HiaBD2/HsfBD2) that are located at opposite ends of the passenger domain and have different binding affinities. Interestingly, in both cases, the binding domain at the C-terminal end of the protein is associated with greater affinity. The work presented here suggests that the difference in affinity is most likely explained by changes in the composition of the binding pocket identified in HiaBD1.^18^ Thus, due to their trimeric structure, Hia and Hsf display a multivalent binding site with six subsites (three HiaBD1/HsfBD1 subsites and three HiaBD2/HsfBD2 subsites). As outer-membrane adhesins, the functions of Hia and Hsf are to make contact with host cell receptors and to facilitate interaction between the bacterium and the host. To achieve this goal, the adhesin must be long enough to extend beyond the lipopolysaccharide layer in the outer membrane of the bacterium and to penetrate the glycan layer surrounding the host cell. In addition, the adhesin must be flexible in order to form an intimate interaction with the host and to surmount mechanical forces in the respiratory tract. Based on the available structures of Hia_51–166_ (∼ 53 Å long), Hia_307–422_ (∼ 75 Å long), Hia_541–705_ (∼ 80 Å long), and Hia_973–1098_ (∼ 92 Å long), the estimated length of the Hia passenger domain is at least 600 Å. The estimated length of Hsf is at least 1200 Å. Because Hsf is present in encapsulated strains of *H. influenzae*, this adhesin may indeed need to be longer to reach beyond the capsule.

The repetitive domain architecture and the two groups of binding pockets in Hia and Hsf suggest a two-step mechanism for host recognition and attachment (Fig. 6b). We propose that the distal binding domain (HiaBD2/HsfBD2) initiates the initial interaction with the host cell surface. Subsequently, the repetitive domain architecture between the two adhesive domains, reminiscent of the repetitive Ig-fold architectures of intimin or P and type 1 pili,^23–25^ provides the flexibility required to allow the HiaBD1/HsfBD1 domain to compete with HiaBD2/HsfBD2 for the very same receptor or to interact with a second receptor on the host cell surface. Via this two-step mechanism, the bacterium is pulled closer towards the host cell and forms an intimate association that is strong enough to surmount the mechanical defensive force in the host.

The vast size and modular architecture of Hia and Hsf must have an impact on their mechanism of secretion through the outer membrane. To date, two secretion models appear to be consistent with the known structural biology of trimeric autotransporters: the so-called “hairpin” and “Omp85” models. Recent advances in the structural biology of the DegP chaperone suggest a variation in the Omp85 model in which DegP forms a hydrophobic cage vast enough to accommodate folded outer-membrane proteins, captures these proteins in the periplasm, and then delivers them to the Omp85/YaeT complex for insertion into the outer membrane.^26^ Folding of membrane proteins within the periplasm has been suggested by studies of PhoE,^27^ EspP,^28^ and Hia.^16^ One objection to this mechanism being applied to Hia and Hsf is that these proteins are too long to be accommodated within the DegP cage. However, it is possible that the DegP cage surrounds only the translocator domain and carries out the periplasmic shuttling function, with the folded hydrophilic passenger domain protruding from the cage.

## Materials and Methods

### Bacterial strains, plasmids, and culture conditions

Laboratory strains employed in these studies include *Escherichia coli* B834(DE3) (methionine auxotroph; Novagen), *E. coli* DH5α (Life Technologies), and *E. coli* BL21(DE3). *E. coli* strains were grown in Luria–Bertani (LB) agar or in LB and stored at − 80 °C in LB with 50% glycerol. Antibiotic concentrations used to select for plasmids included 100 μg ml^− 1^ ampicillin and 12.5 μg ml^− 1^ tetracycline.

### Construction of plasmids used in this study

To generate constructs for crystallography experiments, *H. influenzae* genomic DNA was used as template to amplify the coding sequence for Hia residues 51–166, 307–422, and 937–1098, with either a BamHI site and an NdeI site or two BsaI sites at the ends of the forward and reverse primers, respectively. The primers were as follows:

5′-GACGACATATGAACAATACTCCTA TTACGAATAAG-3′ (forward primer; Hia_51–166_)
5′-GACGAGGATCCTTACGCTAAAGCAAAGGTAATGGTGTG-3′ (reverse primer; Hia_51–166_)
5′-GACGACATATGAAAGAAAACGGTAAGAGAACGAA-3′ (forward primer; Hia_307–422_)
5′-GACGAGGATCCTTATTTCGCATCATACTTAACGGTAAT-3′ (reverse primer; Hia_307–422_)
5′-ATGGTAGGTCTCACTCCGACGGTACGGCTGATATGACCA-3′ (forward primer; Hia_937–1098_)
5′-ATGGTAGGTCTCATATCACCACTGGTAACCAACACCTGCTG-3′ (reverse primer; Hia_937–1098_).

The resulting PCR fragments encoding Hia_51–166_ and Hia_307–422_ were digested with BamHI and NdeI and ligated into BamHI/NdeI-digested pET15b (Novogen), generating pET15b-Hia_51–166_ and pET15b-Hia_307–422_. In order to obtain experimental phases for structural determination, a point mutation in Hia_307–422_ converting the Ile at position 324 into Met was generated using primers 5′-ACTTCTGTTATGAAAGAAAAAGAC-3′ (forward) and 5′-GTCTTTTTCTTCATAACAGAAGT-3′ (reverse), and the QuickChange™ site-directed mutagenesis kit (Stratagene). (The base mutation responsible for the amino acid is underlined.)

To generate a plasmid to overexpress Hia_937–1098_ in the outer membrane of *E. coli*, the OmpA signal peptide encoded in pASK-IBA12 (IBA) was used. The PCR fragment of Hia_937–1098_ was digested with BsaI and ligated into BsaI-digested pASK-IBA12, generating pASK-Hia_937–1098_.

### Expression, purification, and crystallization

*E. coli* BL21(DE3)/pET15b-Hia_51–166_ and methionine auxotroph *E. coli* B834(DE3)/pET15b-Hia_307–422_I324M were grown at 37 °C to an OD_600_ of 0.5 and then induced with 1 mM IPTG for 4 h. Selenomethionine-derivatized Hia_307–422_ was expressed in cells grown in SelenoMet Medium Base (AthenaES) supplemented with SelenoMet Nutrient Mix (AthenaES) and 40 mg l^− 1^ seleno-l-methionine (Acros). Following induction, bacteria were centrifuged at 4000*g* for 20 min, and cell pellets were resuspended in 20 mM sodium phosphate (pH 7.4), 500 mM NaCl, and 40 mM imidazole. Bacteria were disrupted with a high-pressure homogenizer (Glen Creston Ltd.). The clear lysate (30,000*g* for 30 min) was loaded onto a HisTrap column (GE Healthcare) and eluted with 20 mM sodium phosphate (pH 7.4), 500 mM NaCl, and 500 mM imidazole. The eluate was pooled and incubated with thrombin protease (Novagen) at room temperature for 10 h and then dialyzed against 20 mM Tris (pH 8.0) and 20 mM NaCl at 4 °C for 10 h. Subsequently, the protein sample was loaded back onto a HisTrap column, and the flow-through was loaded onto a Q-Sepharose column. A linear salt gradient (pH 8.0; 0–1 M) was used for elution. The peak fractions (∼ 0.2 M for Hia_51–166_ and ∼ 0.3 M for Hia_307–422_) were pooled, and purity was further polished by an S100 gel-filtration column. The final purified protein sample was estimated to be ∼ 95% pure by Coomassie-blue-stained SDS-PAGE. The eluate of the S100 was subsequently concentrated to ∼ 47 mg/ml for Hia_51–166_ and to ∼ 62 mg/ml for Hia_307–422_ using a Centricon concentrator with a 10-kDa cutoff (Amicon).

To purify Hia_937–1098_, *E. coli* BL21(DE3)/pASK-Hia_937–1098_ was grown at 37 °C and induced with 0.2 mg l^− 1^ anhydrotetracycline (IBA) for 14 h. Following induction, bacteria were centrifuged at 4000*g* for 20 min, and cell pellets were resuspended in 20 mM Tris (pH 8.0) and 150 mM NaCl. Bacteria were disrupted with a high-pressure homogenizer (Glen Creston Ltd.). The membrane proteins were recovered by centrifugation at 30,000*g* for 45 min and solubilized with 5% (wt/vol) Elugent (Calbiochem), 20 mM Tris (pH 8.0), and 150 mM NaCl. The clear lysate (30,000*g* for 45 min) was loaded onto a Strep-Tactin column and eluted with 2.5 mM desthiobiotin, 100 mM Tris (pH 8.0), 150 mM NaCl, 10 mM ethylenediaminetetraacetic acid, and 0.5% (wt/vol) Elugent. The eluate was then incubated with thrombin protease for > 10 h at room temperature before it was dialyzed against 20 mM Tris (pH 8.0), 20 mM NaCl, and 0.5% (wt/vol) Elugent at 4 °C. The resulting protein sample was then reloaded onto a fresh Strep-Tactin column to eliminate the cleaved Strep tag. The flow-through of the Strep-Tactin column was pooled and loaded onto a Q-Sepharose column. A linear salt gradient (pH 8.0; 0–1 M) was used for elution. The peak fractions (∼ 0.2 M) were pooled and dialyzed against 20 mM 4-morpholineethanesulfonic acid (pH 5.2), 20 mM NaCl, and 0.5% (wt/vol) Elugent, and then loaded onto an SP-Sepharose column. A linear salt gradient (pH 5.2; 0–1 M) was used for elution. The peak fraction (∼ 0.5 M) was ∼ 95% pure, as indicated by Coomassie-blue-stained SDS-PAGE. Finally, SP-Sepharose was again used to exchange Elugent with 0.6% (vol/vol) *n*-octyltetraosyethylene (Bachem). The eluate of SP-Sepharose was subsequently concentrated to ∼ 29 mg/ml using a Centricon concentrator with a 10-kDa cutoff (Amicon) and dialyzed against 20 mM 4-morpholineethanesulfonic acid (pH 5.2), 100 mM NaCl, and 0.6% (vol/vol) *n*-octyltetraosyethylene at 4 °C for 14 h.

Hia_51–166_ native crystals, Hia_937–1098_ native crystals, and Hia_307–422_ selenomethionine-derivatized crystals were grown at 20 °C using the hanging-drop vapor-diffusion method. The reservoir solutions are 100 mM Hepes (pH 7.5), 25% polyethylene glycol (PEG) 10,000 (Hia_51–166_), and 100 mM Tris (pH 8.5); 30% PEG 1000 (Hia_307–422_) and 100 mM Hepes (pH 7.0); and 16% PEG 2000 MME (Hia_937–1098_). Crystals of Hia_51–166_ diffracted to 2.0 Å resolution and belonged to space group *I*2_1_2_1_2_1_, with cell dimensions of *a* =  82.2 Å, *b* = 91.0 Å, and *c* = 94.2 Å, and with three molecules in the asymmetric unit. Crystals of selenomethionine-derivatized Hia_307–422_ diffracted to 1.8 Å and belonged to space group *P*6_3_22, with cell dimensions of *a* = 53.9 Å, *b* = 53.9 Å, and *c* = 151.7 Å, and with one molecule in the asymmetric unit. Crystals of Hia_937–1098_ diffracted to 3.0 Å resolution and belonged to space group *C*2, with cell dimensions of *a* = 194.3 Å, *b* = 45.8 Å, *c* = 56.4 Å, and β = 95.1, and with three molecules in the asymmetric unit.

### Data collection and phasing

Diffraction data for Hia_51–166_ and Hia_937–1098_ native crystals were recorded on beamlines ID29 and ID14.1 at the European Synchrotron Radiation Facility (ESRF; Grenoble, France), respectively. MAD diffraction data of selenomethionine-derivatized Hia_307–422_I324M were recorded at selenium edge (0.9785 Å), inflection (0.9790), and remote (0.9750) on beamline ID14.4 at the ESRF. Hia_307–422_I324M MAD data were integrated and scaled using DENZO and SCALEPACK.^29^ MOSFLM/SCALA^30^ was used for Hia_51–166_ and Hia_937–1098_ diffraction data. The statistics of data collection are reported in Table 1.

The MAD method was used to phase Hia_307–422_I324M. One Se position was determined by anomalous-difference Patterson analysis of data with resolutions of between 30 and 3.0 Å, using the program SHELX.^31^ The parameters of the heavy-atom sites were refined using SHARP (Global Phasing).^32^ An interpretable map was obtained after solvent flattening (DM).^30^ The extended phases (1.8 Å) allowed automatic tracing of the backbone (Hia residues 320–422; using the program ARP/wARP^33^), producing a σ_A_-weighted 2*F*_o _− *F*_c_ map of excellent quality into which side chains were built manually using the program COOT^30^ (Supplementary Fig. 1). Hia_51–166_ and Hia_937–1098_ were phased by molecular replacement (PHASER)^30^ using the published structures of Hia_585–705_ and Hia_998–1098_, respectively (Protein Data Bank codes 1S7M and 2GR7, respectively) as search models. ARP/wARP and COOT were used as mentioned above to build the model of Hia_51–166_. CNS^34^ and PHENIX.REFINE,^35^ together with intermittent manual building in COOT, were used to build the missing residues (residues 973–997) in Hia_937–1098_. Hia residues 937–972 appear to be disordered, hence the electron density map for these residues is not available for model building.

### Structure refinement

The structure models of Hia_51–166_, Hia_307–422_, and Hia_973–1098_ were refined by conjugate gradient minimization and simulated annealing implemented in PHENIX.REFINE and CNS with intermittent manual rebuilding, refining individual *B*-factors applying TLS correction.^36^ The final model of Hia_51–166_ (*R*/*R*_free_ factors = 19.4/23.6) contains residues 54–165 and 192 water molecules. The final model of Hia_307–422_ (*R*/*R*_free_ factors = 16.2/19.8) contains residues 313–422 and 244 water molecules. The final model of Hia_973–1098_ (*R*/*R*_free_ factors = 23.3/28.5) contains residues 973–1098. The detailed structure refinement statistics are reported in Table 2.

### Accession codes

Coordinates of Hia_51–166_, Hia_307–422_ and Hia_973–1098_ have been deposited to the Protein Data Bank (entry codes 3EMF, 3EMI and 3EMO, respectively).

## Figures and Tables

**Table 1:** Data collection of *H. influenzae* Hia adhesin structures

	Hia_51–166_	Hia_307–422_			Hia_937–1098_
Derivative	Native	Selenomethionine			Native
Dataset		Peak	Inflection	Remote	
Space group (molecules/asymmetric unit)	*I*2_1_2_1_2_1_ (3)	*P*6_3_22 (1)			*C*2 (3)
Unit cell dimensions					
* a* (Å)	82.2	53.9			194.3
* b* (Å)	91.0	53.9			45.8
* c* (Å)	94.2	151.7			56.4
β (°)	95.1				
Source/station^a^	ID29	ID14.4			ID14.1
Wavelength (Å)	0.9729	0.9785	0.9790	0.9750	0.9340
Resolution range (Å)	30–2.0	30–1.8	30–1.8	30–1.8	30–3.0
Observations (*I*/σ(*I*) > 0)	141,856	419,003	419,296	404,071	29,016
Unique reflections (*I*/σ(*I*) > 0)	24,079	12,834	12,852	12,885	8519
Last shell (Å)	2.11–2.00	1.86–1.80	1.86–1.80	1.86–1.80	3.11–3.00
*R*_sym_ (%)b,c	6.5 (31.8)	9.9 (24.2)	9.8 (27.2)	10.5 (28.7)	7.9 (15.0)
Mean (〈*I*/σ(*I*)〉)^c^	19.0 (3.7)	42.0 (11.3)	42.3 (11.3)	42.1 (11.0)	11.1 (4.1)
Completeness (%)^c^	99.3 (95.6)	99.6 (99.9)	99.7 (99.9)	99.7 (100.0)	84.6 (60.8)
Redundancy^c^	5.9 (4.1)	32.6 (28.3)	32.6 (28.2)	31.4 (26.1)	3.6 (2.4)

a Beamline designations refer to the ESRF.

b *R*_sym_ = ∑(*I *− 〈*I*〉)/∑*I*.

c Values for the overall high-resolution are presented shell in parentheses.

**Table 2:** Structure refinement of *H. influenzae* Hia adhesin structures

	Hia_51–166_	Hia_307–422_	Hia_937–1098_
Resolution range (Å)	30–2.0	30–1.8	30–3.0
*R*-factor (%)	19.4	16.2	23.3
*R*-factor (high-resolution shell)^a^	21.3	13.7	29.13
*R*_free_ (%)^b^	23.6	19.8	28.5
*R*_free_ (high-resolution shell)	28.1	19.7	35.2
Total number of nonhydrogen atoms	2720	1030	2617
Protein atoms	2528	786	2617
Water molecules	192	244	0
RMSD^c^			
Bond length (Å)	0.008	0.005	0.006
Bond angle ( °)	1.079	0.881	0.942
Wilson *B*-factor (Å^2^)	30.3	15.8	56.4
Average *B*-factor protein atoms (Å^2^)	44.3	17.8	48.8
Average *B*-factor solvent atoms (Å^2^)	44.4	32.6	
Ramachandran statistics^d^			
Most favored regions (%)	90.9	93.6	82.5
Additionally allowed regions (%)	9.1	6.4	17.1
Generously allowed regions (%)	0	0	0.3
Disallowed regions (%)	0	0	0

a High-resolution shell (see the last shell in Table 1).

b *R*_free_ calculated using 5% or 10% of the total reflections omitted from refinement for Hia_51–166_, Hia_307–422_, and Hia_937–1098_.

c RMSDs from ideal bond lengths and angles between bonded atoms.^37^

d Ramachandran statistics calculated using PROCHECK.^38^

## Footnotes

† http://toolkit.tuebingen.mpg.de/dataa

‡ http://toolkit.tuebingen.mpg.de/dataa/browse

## References

[1] Turk D.C. (1984). The pathogenicity of *Haemophilus influenzae*. J. Med. Microbiol..

[2] Murphy T.F., Bernstein J.M., Dryja D.M., Campagnari A.A., Apicella M.A. (1987). Outer membrane protein and lipooligosaccharide analysis of paired nasopharyngeal and middle ear isolates in otitis media due to nontypeable *Haemophilus influenzae*: pathogenetic and epidemiological observations. J. Infect. Dis..

[3] Spinola S.M., Peacock J., Denny F.W., Smith D.L., Cannon J.G. (1986). Epidemiology of colonization by nontypeable *Haemophilus influenzae* in children: a longitudinal study. J. Infect. Dis..

[4] St Geme J.W. (2002). Molecular and cellular determinants of non-typeable *Haemophilus influenzae* adherence and invasion. Cell. Microbiol..

[5] Barenkamp S.J., St Geme J.W. (1996). Identification of a second family of high-molecular-weight adhesion proteins expressed by non-typeable *Haemophilus influenzae*. Mol. Microbiol..

[6] St Geme J.W., Cutter D., Barenkamp S.J. (1996). Characterization of the genetic locus encoding *Haemophilus influenzae* type b surface fibrils. J. Bacteriol..

[7] Surana N.K., Cutter D., Barenkamp S.J., St Geme J.W. (2004). The *Haemophilus influenzae* Hia autotransporter contains an unusually short trimeric translocator domain. J. Biol. Chem..

[8] Cotter S.E., Yeo H.J., Juehne T., St Geme J.W. (2005). Architecture and adhesive activity of the *Haemophilus influenzae* Hsf adhesin. J. Bacteriol..

[9] Cotter S.E., Surana N.K., St Geme J.W. (2005). Trimeric autotransporters: a distinct subfamily of autotransporter proteins. Trends Microbiol..

[10] Linke D., Riess T., Autenrieth I.B., Lupas A., Kempf V.A. (2006). Trimeric autotransporter adhesins: variable structure, common function. Trends Microbiol..

[11] Henderson I.R., Navarro-Garcia F., Desvaux M., Fernandez R.C., Ala'Aldeen D. (2004). Type V protein secretion pathway: the autotransporter story. Microbiol. Mol. Biol. Rev..

[12] Dautin N., Bernstein H.D. (2007). Protein secretion in Gram-negative bacteria via the autotransporter pathway. Annu. Rev. Microbiol..

[13] Jacob-Dubuisson F., Fernandez R., Coutte L. (2004). Protein secretion through autotransporter and two-partner pathways. Biochim. Biophys. Acta..

[14] Roggenkamp A., Ackermann N., Jacobi C.A., Truelzsch K., Hoffmann H., Heesemann J. (2003). Molecular analysis of transport and oligomerization of the *Yersinia enterocolitica* adhesin YadA. J. Bacteriol..

[15] Oomen C.J., van Ulsen P., van Gelder P., Feijen M., Tommassen J., Gros P. (2004). Structure of the translocator domain of a bacterial autotransporter. EMBO J..

[16] Meng G., Surana N.K., St Geme J.W., Waksman G. (2006). Structure of the outer membrane translocator domain of the *Haemophilus influenzae* Hia trimeric autotransporter. EMBO J..

[17] Laarmann S., Cutter D., Juehne T., Barenkamp S.J., St Geme J.W. (2002). The *Haemophilus influenzae* Hia autotransporter harbours two adhesive pockets that reside in the passenger domain and recognize the same host cell receptor. Mol. Microbiol..

[18] Yeo H.J., Cotter S.E., Laarmann S., Juehne T., St Geme J.W., Waksman G. (2004). Structural basis for host recognition by the *Haemophilus influenzae* Hia autotransporter. EMBO J..

[19] Szczesny P., Lupas A. (2008). Domain annotation of trimeric autotransporter adhesins—daTAA. Bioinformatics.

[20] Nummelin H., Merckel M.C., Leo J.C., Lankinen H., Skurnik M., Goldman A. (2004). The *Yersinia* adhesin YadA collagen-binding domain structure is a novel left-handed parallel beta-roll. EMBO J..

[21] Hoiczyk E., Roggenkamp A., Reichenbecher M., Lupas A., Heesemann J. (2000). Structure and sequence analysis of *Yersinia* YadA and *Moraxella* UspAs reveal a novel class of adhesins. EMBO J..

[22] Szczesny P., Lnke D., Ursinus A., Bär K., Schwarz H., Riess T.M. (2008). Structure of the head of the *Bartonella* adhesin BadA. PLoS Pathog..

[23] Luo Y., Frey E.A., Pfuetzner R.A., Creagh A.L., Knoechel D.G., Haynes C.A. (2000). Crystal structure of enteropathogenic *Escherichia coli* intimin–receptor complex. Nature.

[24] Sauer F.G., Futterer K., Pinkner J.S., Dodson K.W., Hultgren S.J., Waksman G. (1999). Structural basis of chaperone function and pilus biogenesis. Science.

[25] Choudhury D., Thompson A., Stojanoff V., Langermann S., Pinkner J., Hultgren S.J., Knight S.D. (1999). X-ray structure of the FimC–FimH chaperone–adhesin complex from uropathogenic *Escherichia coli*. Science.

[26] Krojer T., Sawa J., Schafer E., Saibil H.R., Ehrmann M., Clausen T. (2008). Structural basis for the regulated protease and chaperone function of DegP. Nature.

[27] Eppens E.F., Nouwen N., Tommassen J. (1997). Folding of a bacterial outer membrane protein during passage through the periplasm. EMBO J..

[28] Ieva R., Skillman K.M., Bernstein H.D. (2008). Incorporation of a polypeptide segment into the beta-domain pore during the assembly of a bacterial autotransporter. Mol. Microbiol..

[29] Otwinowski Z., Minor W. (1997). Processing of X-ray diffraction data collected in oscillation mode. Methods Enzymol..

[30] CCP4 (1994). The CCP4 suite: programs for protein crystallography. Acta Crystallogr. Sect. D.

[31] Sheldrick G.M., Schneider T.R. (1997). SHELXL: high-resolution refinement. Methods Enzymol..

[32] delaFortelle E., Bricogne G. (1997). Maximum-likelihood heavy-atom parameter refinement for multiple isomorphous replacement and multiwavelength anomalous diffraction methods. Methods Enzymol..

[33] Lamzin V.S., Wilson K.S. (1993). Automated refinement of protein models. Acta Crystallogr. Sect. D.

[34] Brunger A.T., Rice L.M. (1997). Crystallographic refinement by simulated annealing: methods and applications. Methods Enzymol..

[35] Afonine P.V., Grosse-Kunstleve R.W., Adams P.D. (2005). CCP4 Newsl..

[36] Winn M.D., Isupov M.N., Murshudov G.N. (2001). Use of TLS parameters to model anisotropic displacements in macromolecular refinement. Acta Crystallogr. Sect. D.

[37] Engh R.A., Huber R. (1991). Accurate bond and angle parameters for x-ray protein structure refinement. Acta Crystallogr..

[38] Laskowski R.A., MacArthur M.W., Moss D.S., Thornton J.M. (1993). PROCHECK: a program to check the stereochemical quality of protein structures. J. Appl. Cryst..

