# Supplementary material for: Repetitive Architecture of the Haemophilus influenzae Hia Trimeric Autotransporter
Source: J Mol Biol. 2008 Dec 26;384(4):824–36. doi: 10.1016/j.jmb.2008.09.085 (PMC2597055; doi:10.1016/j.jmb.2008.09.085)

**Supplementary materials**

**Legend to supplementary figures**

**Supplementary Figure 1. Electron density maps of Hia_51-166_, Hia_307-422_ and Hia_973-1098_.** The σ_A_-weighted 2*F_o_*-*F_c_* electron densities of the putative host cell receptor binding site around Q82 in Hia_51-166_ (A), around W373 in Hia_307-422_ (B) and the Neck-forming loop in Hia_973-1098_ (C) are contoured at 1 σ level.

**Supplementary Figure 2. The architectures of trimeric autotransporters with multi repetitive Trp-ring domains arrangement predicted by the daTAA server.** From left to right: *Neisseira meningitis* NhhA adhesin, non-capsulated *H.influenzae* Hia adhesin, encapsulated *H.influenzae* Hsf adhesin, *Actinobacillus actinomycetemcomitans* EmaA adhesin*, Pasteurella multocida* hsf adhesin, *Xylella fastidiosa* surface protein XF1981, *Xylella fastidiosa* surface protein XF1529. The terminology for each domain is defined in <http://toolkit.tuebingen.mpg.de/dataa/ browse> (Szczesny and Lupas, 2008).


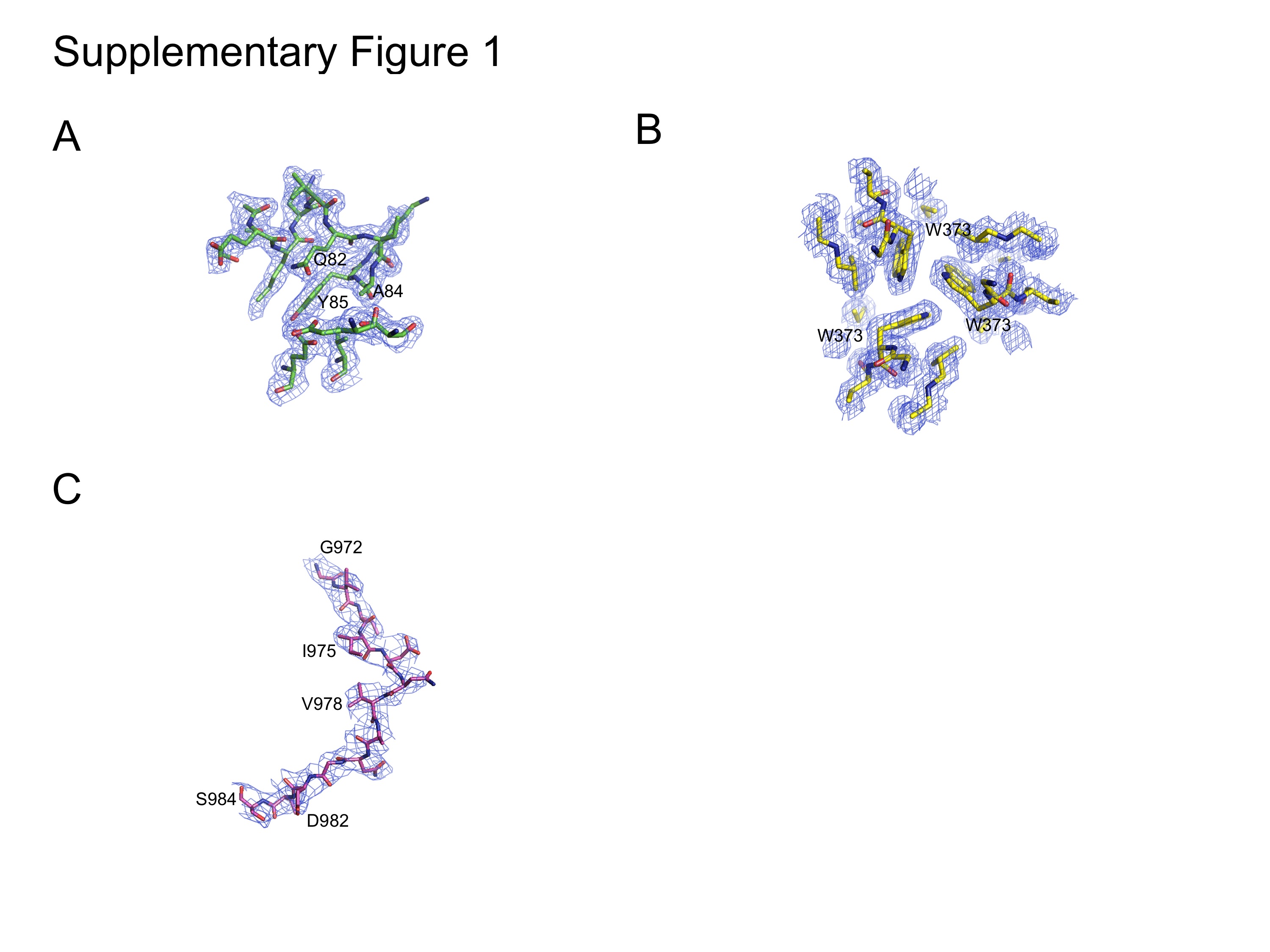


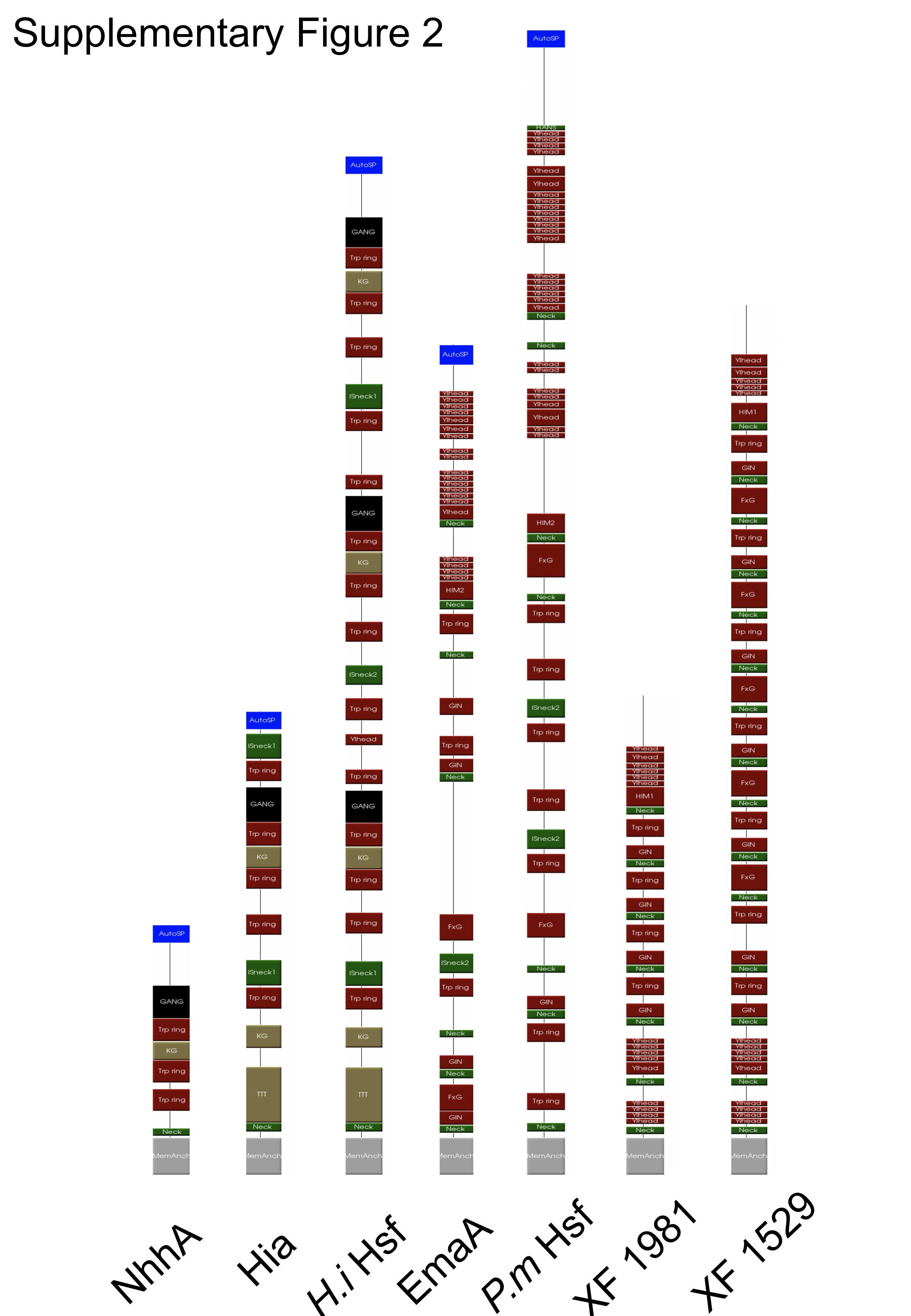

Supplement: Supplementary materials [file mmc1.doc]
